# Supplementary material for: IKKγ/NEMO Is Required to Confer Antimicrobial Innate Immune Responses in the Yellow Mealworm, Tenebrio Molitor
Source: Int J Mol Sci. 2020 Sep 14;21(18):6734. doi: 10.3390/ijms21186734 (PMC7555931; doi:10.3390/ijms21186734)
Supplement: Supplementary file 1 [file ijms-21-06734-s001.pdf]

International Journal of *Molecular Sciences*

MDPI

Type of the Paper (Article)

IKKγ/NEMO is required to confer antimicrobial innate immune responses in the yellow mealworm, *Tenebrio molitor*

Hye Jin Ko [1#], Yong Hun Jo [1#], Bharat Bhusan Patnaik [2,3], Ki Beom Park [1], Chang Eun Kim [1], Maryam Keshavarz [1], Ho Am Jang [1], Yong Seok Lee [4], and Yeon Soo Han [1*]

[1] Department of Applied Biology, Institute of Environmentally-Friendly Agriculture (IEFA), College of Agriculture and Life Sciences, Chonnam National University, Gwangju 61186, Republic of Korea; H.J.K (hjngo0129@naver.com), Y.H.J (yhun1228@jnu.ac.kr), K.B.P (misson112@naver.com), C.E.K (chang9278@naver.com), M.K (Mariakeshavarz1990@gmail.com), H.A.J (hoamjang@gmail.com), Y.S.H (hanys@jnu.ac.kr)

[2] School of Biotech Sciences, Trident Academy of Creative Technology (TACT), Chandrasekharpur, Bhubaneswar, Odisha, 751024, India; B.B.P (drbharatbhusan4@gmail.com)

[3] P.G. and Research Department of Bio-sciences and Bio-technology, Fakir Mohan University, Nuapadhi, Balasore, Odisha, 756089, India; B.B.P (drbharatbhusan4@gmail.com)

[4] School of Biotechnology and Life Sciences, College of Natural Sciences, Soonchunhyang University, 22 Soonchunhyangro, Shinchang-myeon, Asan, Chungchungnam-do 31538, South Korea; Y.S.L (yslee@sch.ac.kr)

* Correspondence: hanys@jnu.ac.kr; Tel.: +82-62-530.2072

[#] These authors contributed equally to this work

Received: date; Accepted: date; Published: date

Supplementary figures

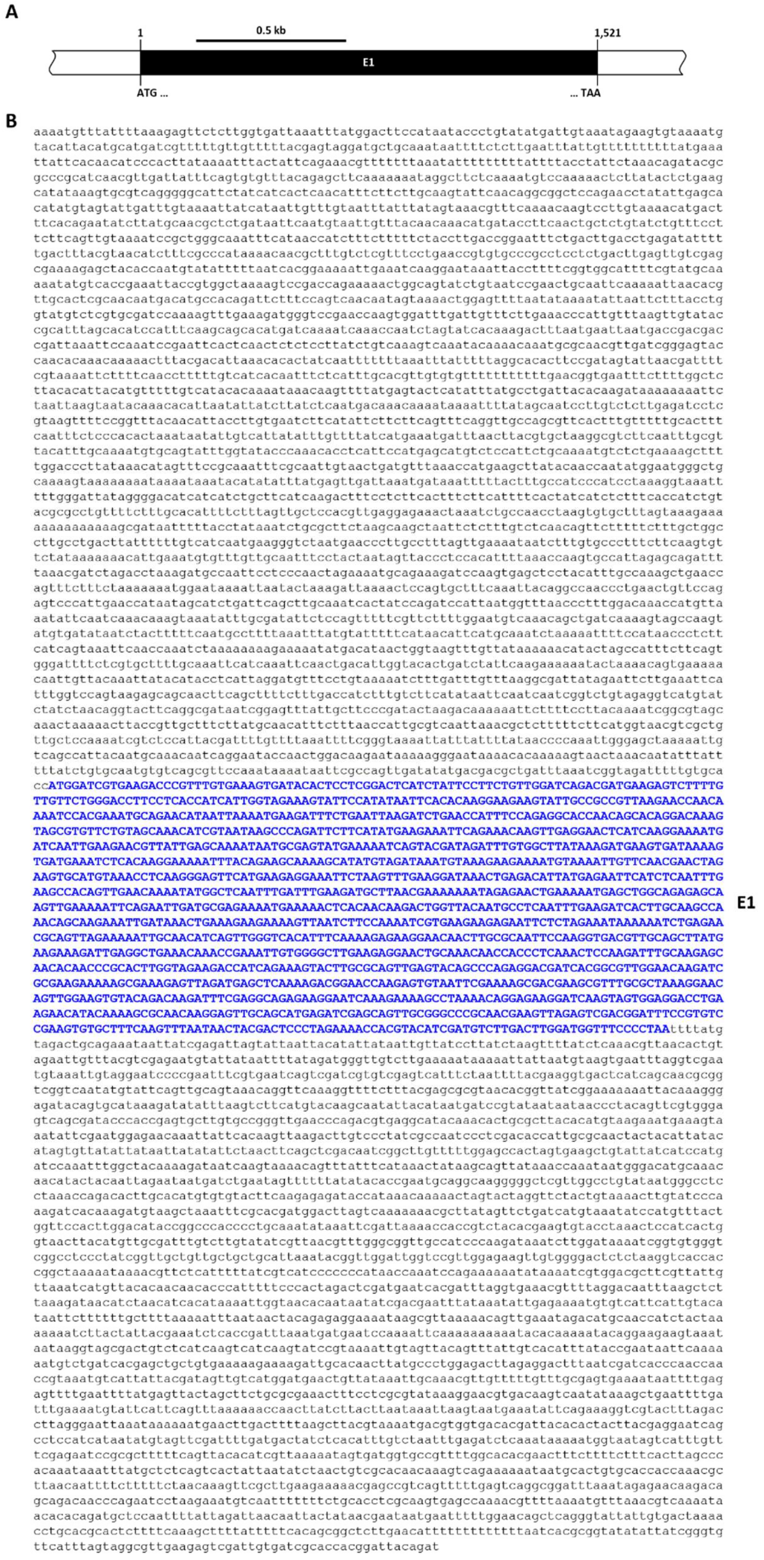

Figure S1. Gene organization of *Tenebrio molitor IKKγ* (*Tm*IKKγ). (A) The gene is composed of a single exon (E1) represented by a coding region of 1,521 nucleotides. (B) The gene sequence (coding region) of *TmIKKγ* is depicted in blue (capital font).

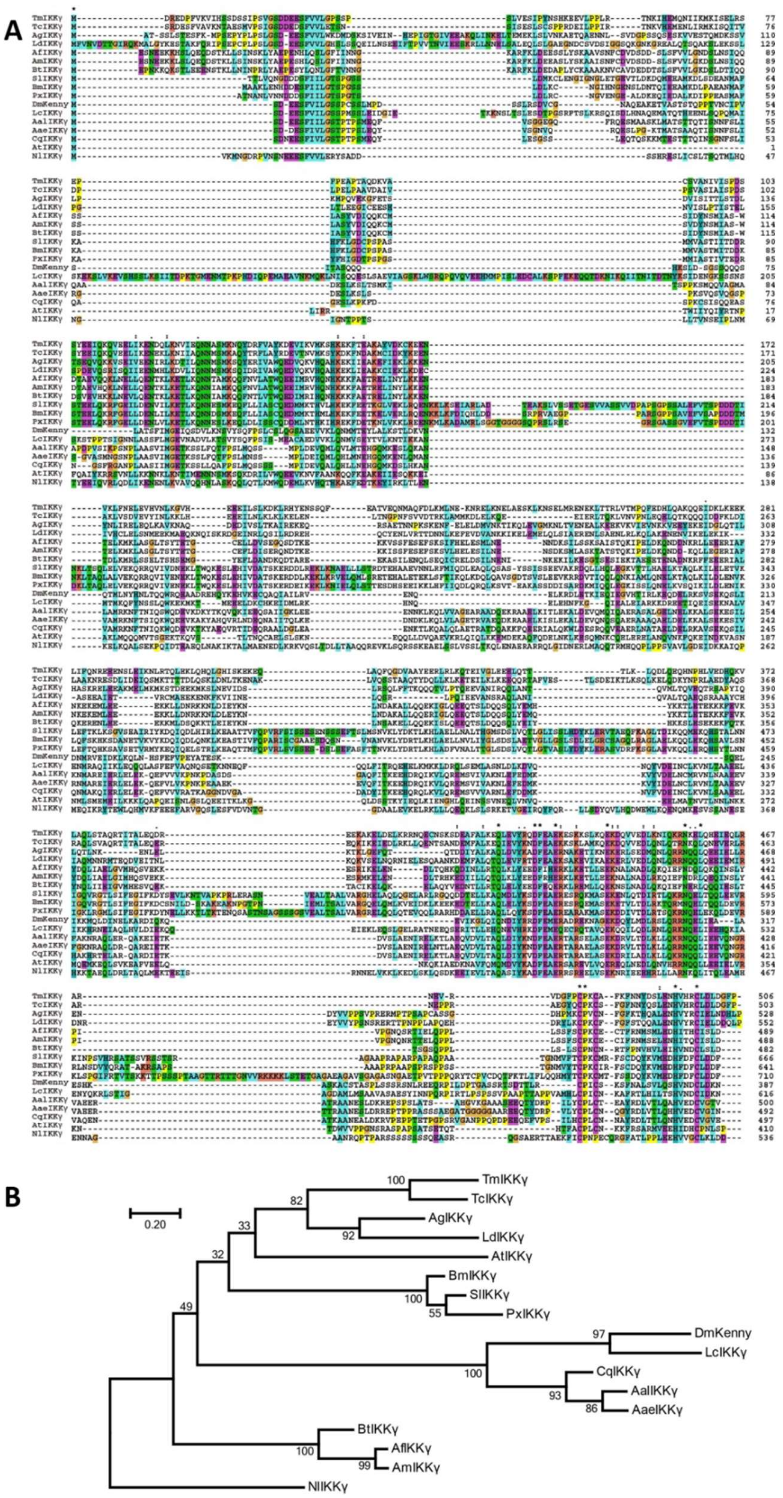

Figure S2. Primary sequence analysis of *Tm*IKKγ at the amino acid sequence level. (A) Multiple sequence alignment of the full-length *Tm*IKKγ with IKKγ protein from other insects. The alignment was conducted using Clustal X (version 2.1). (B) Phylogenetic analysis of *Tm*IKKγ with select insect IKKγ. A bootstrap consensus tree was constructed using MEGA 7 using the neighbor-joining method. The percentage of trees in which the associated taxa clustered together is shown next to the branches. The GeneBank accession numbers of the analyzed IKKγ protein sequences are as follows: *Dm*Kenny,

Drosophila melanogaster Kenny isoform A (NP_523856.2); *Tc*IKKγ, *Tribolium castaneum* IKKγ (EEZ99211.1); *Ag*IKKγ, *Anoplophora glabripennis* NEMO isoform X2 (XP_018567507.1); *Ld*IKKγ; *Leptinotarsa decemlineata* NEMO isoform X2 (XP_023020712.1); *At*IKKγ, *Aethina tumida* NEMO isoform X3 (XP_019868905.1); *Cq*IKKγ, *Culex quinquefasciatus* NEMO (XP_001848522.1); *Lc*IKKγ, *Lucilia cuprina* NEMO isoform X2 (XP_023307105.1); *Aal*IKKγ, *Aedes albopictus* NEMO-like (XP_019530823.1); *Aae*IKKγ, *Aedes aegypti* NEMO (EAT35311.1); *Px*IKKγ, *Papilio xuthus* NEMO (KPI94552.1); *Sl*IKKγ, *Spodoptera litura* NEMO isoform X2 (XP_022838009.1); *Bm*IKKγ, *Bombyx mori* NEMO isoform X2 (XP_004924233.1); *Bt*IKKγ, *Bombus terrestris* NEMO isoform X2 (XP_020721792.1); *Af*IKKγ; *Apis florea* NEMO-like (XP_003694538.1), *Am*IKKγ, *Apis mellifera* NEMO (XP_001120619.2); *Nl*IKKγ, *Nilaparvata lugens* NEMO (XP_022185025.1).
